# Supplementary material for: Stakeholders engagement for solving mobility problems in touristic remote areas from the Baltic Sea Region
Source: PLoS One. 2021 Jun 23;16(6):e0253166. doi: 10.1371/journal.pone.0253166 (PMC8221474; doi:10.1371/journal.pone.0253166)
Supplement: S1 Appendix — (DOCX) [file pone.0253166.s001.docx]

**Appendix 1**

Basic information about the pilot regions and the indicators of transport accessibility

| Region (Country) | Area of the region (km2) | Total Population | Population change per 1,000 inhabitants (2014-2019) | Density of population (number of inhabitants per km2) | Hard paved public roads per 100 km² in km | Cars registered per 1000 inhabitants | Bicycle paths (km) |
| --- | --- | --- | --- | --- | --- | --- | --- |
| Ludwigslust – Parchim municipality; | 4 767 | 211 779 | 0,56 | 45 | 129,7 | 581 | n/a |
| Mecklenburg-Western Pomerania  (Federal State, Germany) | 23 294 | 1 608 138 | - 0,40 | 69 | 126,16 | 538 | 2595 |
| Vidzeme Region (Latvia) | 15 257 | 183 938 | -89 | 12 | 18,7 | 423,8 | < 0,5 km per 1000 inhabitants |
| Birštonas municipality; | 124 | 4 117 | - 57,7 | 33,7 | 72,6 | 549 | 20,3 |
| Druskininkai municipality (Lithuania) | 453,9 | 19 360 | - 75,6 | 42,7 | 47,6 | 518 | 65,6 |
| Setesdal Region (Norway) | 5 519 | 7 857 | 0,45 | 1,42 | 11,77 | 0,56 | 38 |
| Hajnówka district (Poland) | 1 624 | 42 610 | -12,4 | 27 | 39,5 | 571 | 652 |
| Zaonezhsky peninsula of Medvezhyegorsk District (Republic of Karelia, Russia) | 12 000 | 3432* | n/a | 0,3 (2,0 - Medvezhyegorsk district) | n/a | n/a | n/a |

*in 3 rural settlements (Velikaya Guba - 1597, Tolvuya rural settlement -1066, Shun’ga – 769) = pilot region

Source: Regional Action Plans, 2020 (internal project documentation).
