# Supplementary material for: Stakeholders engagement for solving mobility problems in touristic remote areas from the Baltic Sea Region
Source: PLoS One. 2021 Jun 23;16(6):e0253166. doi: 10.1371/journal.pone.0253166 (PMC8221474; doi:10.1371/journal.pone.0253166)
Supplement: S2 Appendix — (DOCX) [file pone.0253166.s002.docx]

**Appendix 2**

Pred-defined categories of content used in the expert workshops including support questions and manual during regional stakeholder involvement strategies elaboration (Table 1-4 Precoding categories of content)

Table 1. List of stakeholders’ groups important from the point of view of solving mobility problems

| Stakeholders’ groups |
| --- |
| (1) local and regional authorities (2) local residents, (3) vistors, (4) local and regional mobility service (eg. taxi services, car renting), (5) local and regional transportation planners, (6) local and regional land-use planners |

Table 2. Levels of influence stakeholder group to solve mobility problems

| Assessment | Level of influence | Definition |
| --- | --- | --- |
| 1 | Low | The stakeholder has very little, if any, influence on the project |
| 2 | Rather low | The stakeholder has some influence on the project |
| 3 | Medium | The stakeholder has an influence on the project |
| 4 | High | The stakeholder has a high influence on the project |
| 5 | Very high | The stakeholder has a significant influence on the project |

Table 3. Levels of relevance stakeholder group to solve mobility problems

| Assessment | Level of relevance | Definition |
| --- | --- | --- |
| 1 | Low | Stakeholder has very little or no interest in the project / The project has very little or no impact on the stakeholder |
| 2 | Rather low | The stakeholder has little interest in the project / the project has little impact on the stakeholder |
| 3 | Medium | Stakeholder is interested in the project / The project has an impact on the stakeholder |
| 4 | High | Stakeholder is clearly interested in the project / The project has a high impact on the stakeholder |
| 5 | Very high | Stakeholder is very interested in the project / The project has a significant impact on the stakeholder |

Table 4. Levels of engagement stakeholder group to solve mobility problems

| Level of engagement | Type | Definition |
| --- | --- | --- |
| A | Information | Providing citizens with knowledge, information on specific problems and proposals for their solutions |
| B | Consultation | Organizing the process of two-way communication between authority-citizen, citizen-authority, as well as readiness to apply specific solutions, take comments |
| C | Involvement | Taking into account comments, opinions, solutions or their elements in the created, implemented or monitored public policies |
| D | Collaboration | Involvement of social, economic or citizen partners at every stage of the decision-making process |
| E | Empowerment | Handing over the final decision to the citizens |
