# Supplementary material for: Stakeholders engagement for solving mobility problems in touristic remote areas from the Baltic Sea Region
Source: PLoS One. 2021 Jun 23;16(6):e0253166. doi: 10.1371/journal.pone.0253166 (PMC8221474; doi:10.1371/journal.pone.0253166)
Supplement: S3 Appendix — (DOCX) [file pone.0253166.s003.docx]

**Appendix 3**

Stakeholders influence on the project identified in regional stakeholder involvement strategies

| Country | Main stakeholders group | | | | | | | | | | | | | | | | | |
| --- | --- | --- | --- | --- | --- | --- | --- | --- | --- | --- | --- | --- | --- | --- | --- | --- | --- | --- |
|  | Residents | | | Authorities | | | | Business/operators/services | | | | | Visitors | | Others | | | |
|  | Residents | Young residents | Users | Local authority | Regional authority | Local spatial planners | Regional spatial planners | Transportation company | Service providers | Tourist companies | Regional business | Local business | Summer dwellers | Tourists | Researchers & experts | Museums | NGOs | Rescue services |
| Poland | 4 | 5 | 3 | 4 | 5 | - | 0 | 5 | - | - | - | 4 | - | - | - | - | - | - |
| Latvia | 5 | - | - | 5 | - | - | - | - | 5 | - | - | - | 4 | 4 | 2 | - | - | - |
| Lithuania | 3 | - | - | 3 | - | - | - | - | - | - | 5 | - | - | 3 | - | - | - | - |
| Russia | 3 | - | - | 3 | 5 | - | - | - | - | 2 | - | 4 | - | 4 | 3 | 4 | 2 | 4 |
| Norway | 3 | 3 | - | 5 | 5 | 4 | 4 | 5 | - | 4 | - | - | - | 3 | - | - | - | - |
| Germany | 4 | - | - | 5 | 4 | - | - | 5 | 4 | - | - | - | - | - | 3 | - | - | - |
