# Supplementary material for: Stakeholders engagement for solving mobility problems in touristic remote areas from the Baltic Sea Region
Source: PLoS One. 2021 Jun 23;16(6):e0253166. doi: 10.1371/journal.pone.0253166 (PMC8221474; doi:10.1371/journal.pone.0253166)
Supplement: S5 Appendix — (DOCX) [file pone.0253166.s005.docx]

**Appendix 5**

Experts opinions about stakeholders’ groups, level of its engagement and tools of engagement identified in regional stakeholder involvement strategies

| Country, region | Key stakeholders’ groups | Level of engagement* | Tools |
| --- | --- | --- | --- |
| Poland, Hajnowka district | Local/regional residents (divided into residents, young residents, bikes users) | A,B,C,D | Online surveys, workshops, meetings, community forum, face-to-face meetings |
|  | Local authorities | C, D, E | Meetings with inhabitants, face-to-face meetings, workshops |
|  | Regional authority | C, D, E | Exploratory field visits, face-to-face meetings, study visits |
|  | Transportation companies | C,D | Working party, joint piloting, workshops |
|  | Local businesses | C,D | Individual interviews, participatory observation, meetings with the authorities, online surveys |
| Latvia, Vidzeme | Local residents | C | Focus groups meetings especially with vulnerable groups |
|  | Local authorities, social services | D | Meetings and workshops with service providers |
|  | Summer dwellers | C | Informing through media and internet |
|  | Tourists | C |  |
|  | Tourism specialists | D | Collaboration in testing activity |
|  | E-bike service providers | D | Collaboration in testing activity, workshop with local authorities |
| Lithuania, Birštonas and Druskininkai | Local residents | B | Meetings, questionnaires, media and internet campaign, electronic and paper surveys |
|  | Tourists | B |  |
|  | Local authorities | C | Meetings, demonstration of the results, discussion |
|  | Service provider in the region | B | Informing about the project, interactive workshop about mobility needs |
| Russia, Zaonezhye | Local residents | A,C | Meetings, information, discussions, field trip, pools |
|  | Local/ district authorities | A,C,D | Meetings, information, discussions of the plans and needs |
|  | Regional authority | A,E | Talks both personal and with small groups |
|  | Local businesses | A,C | Meetings, information, discussions of the plans and needs |
|  | Kizhi State museum-reserve | D | Develop activities together |
|  | Tourists | A | Information, pools |
|  | Tourist companies | C,D | Information, pools |
|  | Rescue services | C,E | Meetings, informing |
|  | Research institutions | C,D | Meetings, informing, discussions of the plans and needs |
|  | NGOs, other institutions | B,C | Meetings, informing |
| Norway, Setesdal | Local residents "all" | B | Media and internet campaign, PPGIS survey |
|  | Local residents "youth" | B |  |
|  | Tourists | A |  |
|  | Tourist operators | D | Informing about results, interactive workshop about the mobility needs |
|  | Local spatial transportation planners | D | Meetings, informing about results, interactive workshops with end-users |
|  | Regional spatial transportation planners | D |  |
|  | Transportation companies | D | Interactive workshop about the mobility needs |
|  | Local politicians | E | Meetings, informing about results |
|  | Regional politicians | E |  |
| Germany, Ludwigslust-Parchim | Local and regional residents | A,B,C | Letter, website, feedback form, suggestion box, workshops, meetings, community forum |
|  | Local authorities | D | The consultative hearings with experts |
|  | Regional authority | D | Working party, workshop, joint piloting |
|  | Transportation operators | D |  |
|  | Public and private service providers | A,B,C | Letter, website, feedback form, suggestion box; workshops, meetings, community forum |
|  | Experts and professionals | B | Workshop, consultative hearing |

* A – information, B – consultation, C – involvement, D – collaboration, E– empowerment.
