# Supplementary material for: Stakeholders engagement for solving mobility problems in touristic remote areas from the Baltic Sea Region
Source: PLoS One. 2021 Jun 23;16(6):e0253166. doi: 10.1371/journal.pone.0253166 (PMC8221474; doi:10.1371/journal.pone.0253166)
Supplement: S6 Appendix — (DOCX) [file pone.0253166.s006.docx]

**Appendix 6**

The 2nd MARA (“Mobility and Accessibility in Rural Areas – New Approaches for Developing Mobility Concepts in Remote Areas”, financed from the Interreg Baltic Sea Region Programme 2014-2020, Priority 3 “Sustainable transport”, Specific objective 3.2 “Accessibility of remote areas and areas affected by demographic change” ) partner meeting, September 11-12, 2019, Hajnowka (Poland)

| 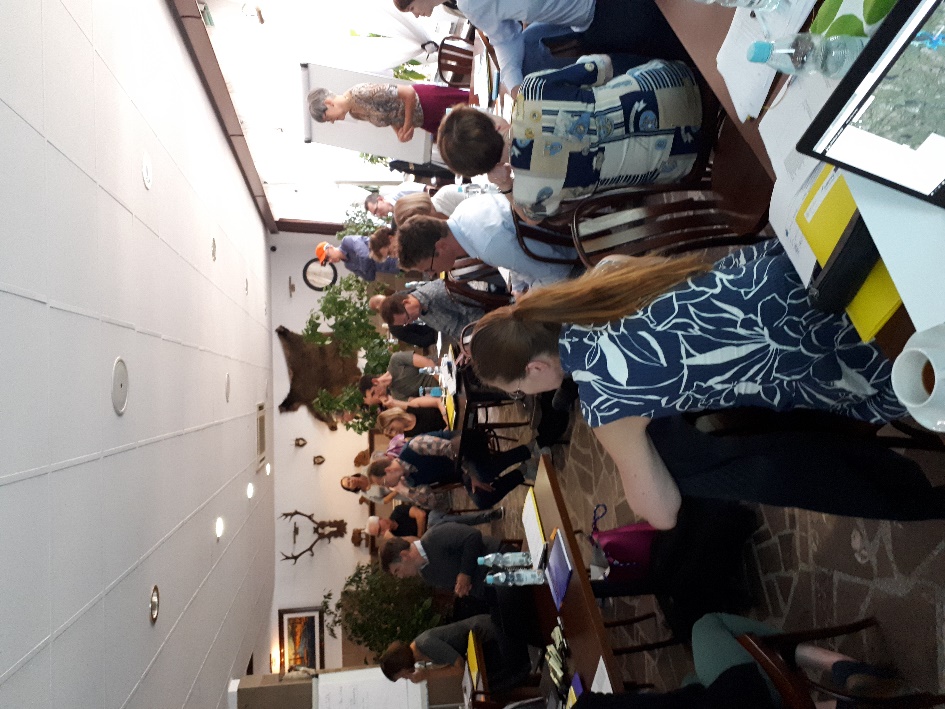 | 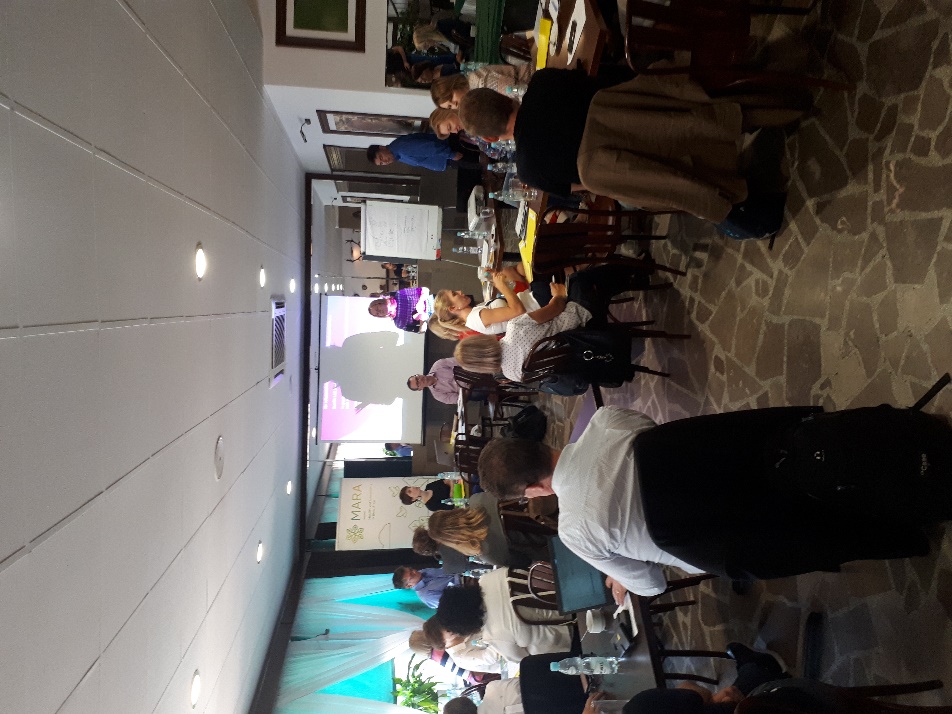 |
| --- | --- |
