# Supplementary material for: Stakeholders engagement for solving mobility problems in touristic remote areas from the Baltic Sea Region
Source: PLoS One. 2021 Jun 23;16(6):e0253166. doi: 10.1371/journal.pone.0253166 (PMC8221474; doi:10.1371/journal.pone.0253166)
Supplement: Attachment — Submitted filename: Response to Reviewers.docx [file pone.0253166.s007.docx]

# **Response to Reviewers**

Title of manuscript:

Stakeholders engagement for solving mobility problems in touristic remote areas from the Baltic Sea Region

The authors wish to thank the editors and the reviewers for their time and effort in reviewing our manuscript. We believe the changes listed have addressed the points raised, making the article suitable for publication.

| **Editor comments:** | **Reply to the review and indication of changes to the text** |
| --- | --- |
| In general, we would expect qualitative studies to include the following:  **1) Defined objectives or research questions. It is important to include research questions that are some of the contributions. These questions should be addressed in the discussion or in conclusions.** | The following three research problems were formulated in the introduction to the research methodology (lines: 282-292).  1. What are the main important groups of stakeholders from the point of view of the possibility of implementing project’s solutions?  2. What methods of stakeholder engagement for solving mobility problems are the most adequate in relation to the identified groups?  3. What are the differences in the selection of these methods depending on the specifics of a given stakeholder group and the level of its engagement? |
| **2) Description of the sampling strategy, including rationale for the recruitment method, participant inclusion / exclusion criteria and the number of participants recruited; Reference authors used to create questionnaires for interviews, pilot testing and validation of interview questionnaires.** | The main criteria of experts’ selection were: the knowledge of local conditions, transportation and mobility expertise, professional activity in the field of issues undertaken in the MARA project, experience in projects with stakeholder involvement. Moreover, the experts represented the diversified groups of stakeholders: local and regional authorities responsible for the local public transport and it's planning and development, business representatives (especially transport operators and service providers), touristic organizations, academic researches, NGOs, innovative transport experts. The experts who met the selection criteria and represented one of the mentioned above group of stakeholders were recruited by MARA project’s partners. The number of experts selected to the study was 40 (fluctuated from 3-4 in Vidzeme Region (Latvia) and Ludwigslust-Parchim district (Germany) to 12 in Hajnowka district (Poland).  The description of the criteria has been extended and made more precise in the text in the chapter Materials and methods (lines: 439-449). |
| **3) Detailed reporting of the data collection procedures; data collection team, dates, problems, etc.** | There were added additional information about data collection procedure lines: 451-463). The experts’ interviews were semi-structured as experts filled in the key stakeholders’ table – Appendix 2. Moreover, the below support questions have been asked to inspire experts to fill in the table.   1. What are the groups of stakeholders important from the point of view of the possibility of implementing project’s solutions? 2. What is the level of influence of stakeholders on the project? 3. What is the level of relevance of the project for the stakeholders (to what extent does the project affect them)? 4. What is the level of engagement of the stakeholders? 5. What are the tools of engagement? What is tools’ attractiveness for the stakeholders? 6. What are the possibilities of tools usage? |
| **4) Data analysis procedures described in sufficient detail to enable replication;** | Data analysis procedures were described in details in Materials and Methods: “The data analysis was made as follows. Firstly, 18 different key stakeholder groups from regional stakeholder engagement strategies were identified. Secondly, in every region the impact of stakeholders on the project (on a scale from 1 to 5) and the importance of the project to stakeholders (on a scale from 1 to 5) assessed by experts were analyzed (an experts’ opinion about stakeholders’ power and influence are presented in Appendix 3 and Appendix 4). The number of strategies in which stakeholder groups were mentioned and average scores of influence and relevance of given group for all six regions were presented. Thirdly, for comparisons, stakeholders were grouped into five main clusters: (1) residents including all kind of residents: permanent, young and older residents, bike users; (2) authorities responsible for case introduction in the region including: local authorities, regional authorities, local spatial planners and regional spatial planners; (3) business and service operators including: transportation companies, service providers, tourist companies, regional business, local business; (4) visitors including: tourists, summer dwellers and (5) others including: researchers & experts, museums, NGO and rescue services. Then, the influence of stakeholders and the relevance of the project in 6 regions were estimated – the average points from all regions and for all stakeholders in cluster were considered. In the next step, the involvement level for five main stakeholders’ clusters in all six regions was presented. The involvement level presents occurrence of experts’ opinions about stakeholders’ level of engagement in every group. In final stage different engagement methods/techniques mentioned in the strategies and assigned for different stakeholders were presented. Experts opinions about stakeholders’ groups and level of its engagement and tools of engagement are gathered in Appendix 5” (lines: 463-485) |
| **5) Content analysis, categories used in the analysis, explanation of the content analysis process and categories, justification of the method.** | We have added the text in the part of Research methodology (lines: 385-389; 392-400):  “The content analysis, carried out for the purposes of the paper, covered six documents under the title "Regional strategy of stakeholders’ engagement in solving of mobility problems". Those documents were developed by members of the MARA project team as part of individual case studies (the above-mentioned areas located in the Baltic Sea region), included in this paper.”  “In the process of analyzing data authors adopted the directed content analysis in which, during the coding process, researchers make use of both codes - formulated on the basis of the existing theory as well as the ones which they develop themselves, relying on obtained results [89]. The choice of the form of the content analysis was due to the fact that the analyzed documents were developed according to one, predetermined scheme and, therefore, contained pre-defined categories of content. However, the authors of the strategy could also propose their own options for the previously prepared scheme of the strategy document”. |
| **6) A discussion of potential sources of bias**; | We have added the text in the part of Research methodology (lines: 401-403):  “The authors of the paper, in carrying out research, tried to demonstrate objectivity (seeking to represent the research material fairly) and sensitivity (responding to even subtle cues to meaning) in the selection and analysis of data from documents” [69]. |
| **7) A discussion of limitations and practical implications.** | There were included in conclusions the information on the scientific contribution and practical implications of the article as well as the main limitations of the research (lines: 758-775, 785-786):  “The conducted research deepens the knowledge on the subject of stakeholder involvement in the management processes of touristic remote areas, in particular in the processes of planning mobility solutions and shaping local policy for sustainable transport. The research findings allow for a better understanding of these processes and support decision-making in the field of methods and techniques of social participation. The implemented research procedure can be helpful for other researchers in designing their own research and the study provides material for comparative analyzes.  Research may also have significant practical implications. By showing examples of good practice on methods of involving different stakeholder groups, they can contribute to the implementation of more effective and sustainable solutions of solving mobility problems in other regions.  The main limitation of the carried out research may be the low representativeness of the results and a certain dose of subjectivism, both in the assessments of experts and in the interpretation of the results by the authors of the paper. Due to the nature of qualitative research, they are more exploratory than conclusive, as it is important to take into account individual contexts and specific cases.”  The authors of the paper, in carrying out research, tried to demonstrate objectivity (seeking to represent the research material fairly) and sensitivity (responding to even subtle cues to meaning) in the selection and analysis of data from documents.  The research findings may also be a starting point for future quantitative research for the authors of the paper. |
| **8) Identification of the case studies, justification of the cases and location map of the case studies.** | As was stated in introduction and added in text (lines: 305-308) that – “The case study analysis is part of the mentioned above transnational project MARA – “Mobility and Accessibility in Rural Areas – New Approaches for Developing Mobility Concepts in Remote Areas””.  Additionally, the location map of the case studies was added in text (Fig. 1) (lines: 318-319)    The selection of case studies resulted from taking into account the specificity of the regions. “All these regions are very attractive to tourists, but they have problems in terms of mobility and communication accessibility, both for residents and tourists. They mainly result from demographic problems (population decline), seasonal fluctuation of tourists, expensive public transport, car dependent lifestyle, car dependent lifestyle, lack of using digital solution” (lines: 311-316). |
| **9) Transcription of the stakeholder interviews (model of interviews in annex and link to the transcription of the interviews)** | The result of the expert opinions was the collection of the completed tables, which template is included in Appendix 2.  The completed tables were gathered in the stakeholder engagement strategy. Cumulated responses are in Appendixes 3, 4.  The aim of the paper was to analyze the content of the participation Strategies, not to conduct interviews with experts. |
| **10) Justification of the identification process of the five stakeholders** | Key stakeholders were grouped into five main groups/clusters. For example, fist group includes all kind of residents: permanent residents, young and older residents, infrastructure users etc. Second group – “Authorities” include all kind of authorities - local authorities, regional authorities, local spatial planners and regional spatial planners. The same is for another groups. The stakeholders were grouped in five clusters to make some overall conclusions as in some regions experts mentioned simply group of residents and in other e.g. "youth" and “other” are mentioned separately.  References to the Appendixes where all stakeholders’ groups in all regions are mentioned have been added in line: 451-453, 667-469, 484-485. |
| **11) Results linked to specific statements from stakeholder interviews** | Results were described linked to Appendix 3, 4 and 5 were an experts’ opinion about stakeholders’ relevance and influence, key stakeholders’ groups and level of its engagement and tools of engagement are gathered. |
