# Supplementary material for: Stakeholders engagement for solving mobility problems in touristic remote areas from the Baltic Sea Region
Source: PLoS One. 2021 Jun 23;16(6):e0253166. doi: 10.1371/journal.pone.0253166 (PMC8221474; doi:10.1371/journal.pone.0253166)
Supplement: Attachment — Submitted filename: Response to Reviewers.docx [file pone.0253166.s008.docx]

# **Response to Reviewers**

Title of manuscript:

Stakeholders engagement for solving mobility problems in touristic remote areas
from the Baltic Sea Region

| **No.** | **Remark/comment** | **Reply to the review and indication of changes to the text** |
| --- | --- | --- |
| 1 | Regarding the research problems, they are not clearly debated in the discussion section. This section needs more depth of analysis. They should also explain why these three research questions have been chosen. | In the paper three research questions (RQ) were stated (In the introduction part, lines: 97-104):  “In order to achieve the intended purpose of the article, three research questions were formulated:  1. What are the main important groups of stakeholders from the point of view of the possibility of implementing project’s solutions?  2. What is the level of engagement of given group of stakeholders on solving the problems of local mobility?  3. What methods of stakeholder engagement for solving mobility problems are the most adequate in relation to the identified groups and desirable level of their involvement?”  The literature review as well as discussion part were corrected and arranged accordingly to the research questions. The corrections made in the literature review clearly indicate why are this research questions were stated.  In the discussion authors indicated links between given RQ and the part of text. They showed that the analysis carried out in the paper allowed to identify and classify key groups of stakeholders and assess their impact on the implementation of the project. It also made it possible to identify and categorize relationships between stakeholders and the methods and techniques of participation used at specific levels of involvement. In the discussion part, the authors pointed out that local government units, when developing their own, long-term strategies for social participation, should adapt the selection of participation methods and techniques to a specific target group and the desired level of their involvement so as to involve stakeholders in the co-decision processes as effectively as possible and achieve effective regional co-management. (lines: 854-859). |
| 2 | Reference authors used to create questionnaires for interviews, pilot testing and validation of interview questionnaires". This question is not answered, these aspects are important to give credibility to your research. | Case study analysis was supported not by the interviews but the structured workshops with the experts who filled in the key stakeholders’ tables answering the support questions, which are presented in the Appendix 2. The gathered materials (filled in tables) where the basis of working out regional stakeholders’ involvement strategies. The research methods were case study analysis and the use of empirical content analysis of documents outlined as “Stakeholder involvement strategy”.  All documents can be found under this link: <https://drive.google.com/drive/folders/1duFIrYTLbKLliz41G9uYIsHf-tr_hjrh?usp=sharing> |
| 3 | They must answer all aspects requested in section 3. "Detailed reporting of the data collection procedures; data collection team, dates, problems, etc." Qualitative research studies should be reported in accordance to the Consolidated criteria for reporting qualitative research (COREQ) checklist or Standards for reporting qualitative research (SRQR) checklist. Further reporting guidelines can be found in the Equator Network's Guidelines for reporting qualitative research. | The interviews were not conducted. In the paper, the research methods were case study analysis and the use of empirical content analysis of documents outlined as “Stakeholder involvement strategy”. So the comment concerning “Detailed reporting of the data collection procedures; data collection team, dates, problems, etc.” is not relevant. |
| 4 | Explain what methods of analysis have been used to achieve the results. Not only the choice of the stakeholders, but the analysis methods used to extract information from the questionnaires. | The information was not extracted from questionnaires. The research use content analysis of secondary data (regional stakeholder involvement strategies) generated as part of the transnational project MARA – “Mobility and Accessibility in Rural Areas – New Approaches for Developing Mobility Concepts in Remote Areas”. The information was extracted from six regional stakeholder involvement strategies elaborated for Vidzeme (Latvia), Birštonas and Druskininkai (Lithuania); Zaonezhye, Karelia (Russia); Setesdal (Norway); Hajnowka district (Poland) and Ludwigslust-Parchim (Germany) regions. Firstly, different key stakeholder groups from regional stakeholder involvement strategies were selected and counted. Secondly, in every region the impact of stakeholders on the project and the importance of the project to stakeholders were analyzed. Then the number of strategies in which stakeholder groups were mentioned and average scores of influence and relevance of given group for all six regions were extracted. Thirdly, for comparisons, stakeholders were grouped into five main clusters and the influence of stakeholders and the relevance of the project in six regions were estimated – the average points from all regions and for all stakeholders in cluster were considered. In the next step, the occurrence of experts’ opinions about stakeholders’ level of engagement in every group was analysed. In final stage different engagement methods/techniques mentioned in the strategies and assigned for different stakeholders were identified and counted (experts’ opinions about stakeholders’ groups and level of its engagement and tools of engagement are gathered in Appendix 5).  The description in text was improved and developed – lines 471-502. Additionally, when describing results – information about the process were added. |
| 5 | Content analysis, categories used in the analysis, explanation of the content analysis process and categories, justification of the method. Where are the analysis categories of your questionnaires and research? They need a read on the categories in qualitative analysis. | The choice of content analysis as a research method was justified. It explained that pre-defined categories of content were used to analyze the content of the stakeholder involvement strategy. These pre-defined categories of content were developed by SYKE (one of the MARA project partners). We have listed them in Annex 1. In the context of the project, they served as guidelines for the development of strategy documents. The authors of the article used them as codes necessary to conduct content analysis.  These pre-defined categories included: (i) a list of potential stakeholder groups, (ii) the possible levels of influence and relevance of a given group of stakeholders for solving mobility problems, (iii) suggested levels of their involvement in improving mobility in a given area (Appendix 2). However, no pre-defined categories of content were proposed in terms of methods / techniques for engaging stakeholders in solving mobility problems.  The description of methodology was improved significantly. |
| 6 | A discussion of potential sources of bias. Some mention is made in the limitations. | The conclusions were supplemented by discussion of potential sources of bias (lines: 9932-944):  “The authors realize that at each stage of the stakeholder analysis, some sources of bias may have occurred [93], resulting both from the formulated research questions, the choice of research areas, and the researchers' approach. A potential source of bias could be the fact that the researchers were an active element of the study and contributed to the Stakeholder involvement strategy in Hajnowka District. However, all the regional strategies analyzed were developed according to uniform guidelines. Potential sources of bias could also appear in the analysis of data and the interpretation of research results, which depends not only on research methods and techniques, but also on the cognitive potential of researchers. "  The limitations include information that the authors tried to reduce subjectivism in carrying out research by demonstrating objectivity (seeking to represent the research material fairly) and sensitivity (responding to even subtle cues to meaning). Thus, they tried to ensure the highest possible quality and the highest value of research. |
| 7 | In relation to the research title and research questions, there should be some more practical implication. | Practical implications have been expanded in the conclusions in relation to the research title and research questions (lines: 914-927)  “Research may also have significant practical implications, in particular for local government units. First of all, they will raise the awareness of decision-makers about the key stakeholder groups and their role in the process of solving the problems of mobility and accessibility of tourism areas and their levels of involvement. They will also allow local governments to better adapt participation tools to specific stakeholder groups, strengthen the levels of involvement of individual groups and undertake more targeted actions in the future. The results of the research can be used as examples of good practice to develop one's own long-term participation strategies in other regions of the Baltic Sea. Such strategies will allow stakeholders to be effectively involved in the co-decision processes and to obtain acceptability of the decisions made. Increasing stakeholders, engagement, will facilitate the development and the implementation of more effective and sustainable solutions of solving mobility problems in other touristic regions. It will also be conducive to the generation of new, creative ideas. " |
| 8 |  | In the editor’s comments there was luck of comment no 8. |
| 9 | Transcription of the stakeholder interviews. Interview transcripts should be included in an attached file. Some of the stakeholder opinions should be integrated into the text to support the discussion, and the research questions; also identify the main stakeholder profiles in relation to the level of influence or engagement. | There was a misunderstanding of the authors' intentions. Not interviews, but workshops were conducted. The authors haven’t gathered the transcription of the stakeholder interviews but they collected filled in tables, which were materials used during workshops. The gathered materials (filled in tables) where the basis of working out regional stakeholders’ involvement strategies. The research methods were case study analysis and the use of empirical content analysis of documents outlined as “Stakeholder involvement strategy”.  The description of methodology was improved significantly. |
| 10 |  | In the editor’s comments there was luck of comment no 10. |
| 11 | Results linked to specific statements from stakeholder interviews. There are no comments from the interviews that provide greater richness to the analysis and plausibility. | There was a misunderstanding of the authors' intentions. Not interviews, but workshops were conducted. The authors haven’t gathered the transcription of the stakeholder interviews but they collected filled in tables, which were materials used during workshops. The gathered materials (filled in tables) where the basis of working out regional stakeholders’ involvement strategies. The research methods were case study analysis and the use of empirical content analysis of documents outlined as “Stakeholder involvement strategy”.  All documents can be found under this link: <https://drive.google.com/drive/folders/1duFIrYTLbKLliz41G9uYIsHf-tr_hjrh?usp=sharing> |
