# Supplementary material for: Stakeholders engagement for solving mobility problems in touristic remote areas from the Baltic Sea Region
Source: PLoS One. 2021 Jun 23;16(6):e0253166. doi: 10.1371/journal.pone.0253166 (PMC8221474; doi:10.1371/journal.pone.0253166)
Supplement: Attachment — Submitted filename: Response to Reviewers.docx [file pone.0253166.s009.docx]

# **Response to Reviewers**

Title of manuscript:

Stakeholders engagement for solving mobility problems in touristic remote areas
from the Baltic Sea Region

The authors wish to thank the editors and the reviewers for their time and effort in reviewing our manuscript. We believe the changes listed have addressed the points raised, making the article suitable for publication.

| **No.** | **Editor comments:** | **Reply to the review and indication of changes to the text** |
| --- | --- | --- |
| 1 | **Clearly explain the contribution of the research (in Introduction).** | The contribution of the research was developed in introduction ((lines: 127-136):  “In the article the concept of collaborative governance and stakeholder involvement methods and techniques are presented. Then materials and methods are provided followed by a discussion of the results and conclusions. The study contributes to the knowledge in several ways. Firstly, it identifies key stakeholder groups in regional stakeholder involvement strategies as well as the level of participation of a given stakeholder group in the process of executing local projects associated with solving the issue of mobility among residents and tourists. Secondly, it points that the level of engaging each group should look different, which depends on the function a given group has in a local transportation project. Thirdly, the selection of engagement techniques should depend on a previously planned, suggested level of including a given stakeholder group in the local project.” |
| 2 | **Include as a secondary objective the practical implication of the research, so that the objectives are connected with the conclusions.** | The practical objective was added in introduction (lines 93-97:  “Practical aims of the research are to raise the awareness of local government units about the key stakeholder groups and their role in the process of solving the problems of mobility and accessibility of tourism areas as well as delivering of guidelines for working out participation strategies.” |
| 3 | **Methodology. You mention that some workshops were held from which information was obtained. Explain the function of these workshops (number, dates, places, information gathering process, etc.), including photographs, if any, are included in the annex.** | The authors of the paper use content analysis of secondary data -six regional stakeholder engagement strategies.  The development of these documents was preceded by a workshop that took place as part of the MARA 2^nd^ partner meeting, on September 11-12, 2019 in Hajnówka (Poland). The workshop was attended by 46 participants representing 12 partners from 9 countries of the Baltic Sea Region: Germany, Poland, Russia, Lithuania, Latvia, Estonia, Finland, Norway, Sweden. The workshop was organized by the County Council in Hajnówka - one of the Polish partners of the project.  **The aim of the workshop**: researchers from the Finnish Environment Institute (SYKE) (one of the MARA project partner) provided guidance for developing stakeholder engagement strategy. The guide prepared by SYKE contained pre-defined categories of content. These categories concerned such issues as: (i) a list of potential stakeholder groups, (ii) possible levels of influence and relevance of a given group of stakeholders for solving mobility-related problems, (iii) suggested levels of their involvement in improving mobility in a given area (Appendix 2). After providing instructions on the strategy development, partners worked on the strategy of involving stakeholders in solving mobility problems in their region. After the workshop, the partners finished their regional stakeholder engagement strategies based on feedback given by SYKE.  <https://www.mara-mobility.eu/11-news/general-news/53-2nd-mara-partner-meeting-took-place-in-hajnowka-poland>  <https://wiz.pb.edu.pl/2019/09/19/mara-2nd-partner-meeting/>  Photos from the workshop are in Attachment 6 |
